# Association of proangiogenic and profibrotic serum markers with lung function and quality of life in sarcoidosis

**DOI:** 10.1371/journal.pone.0247197

**Published:** 2021-02-22

**Authors:** L. Biener, J. Kruse, I. Tuleta, C. Pizarro, M. Kreuter, S. S. Birring, G. Nickenig, D. Skowasch

**Affiliations:** 1 Department of Internal Medicine II–Cardiology, Pneumology and Angiology, University Hospital Bonn, Bonn, Germany; 2 Department of Cardiology I, University Hospital Muenster, Muenster, Germany; 3 Centre for Interstitial and Rare Lung Diseases, Pneumology, Thoraxklinik, University of Heidelberg, Germany and German Centre for Lung Research, Heidelberg, Germany; 4 Centre for Human & Applied Physiological Sciences, School of Basic & Medical Biosciences, Faculty of Life Sciences & Medicine, King’s College London, London, United Kingdom; Virginia Commonwealth University Medical Center, UNITED STATES

## Abstract

**Background:**

Sarcoidosis is a systemic inflammatory granulomatous disease, frequently affecting the lung. If left untreated, it may end in lung fibrosis. Proangiogenic and profibrotic vascular endothelial growth factor (VEGF), transforming growth factor (TGF)-β1, fibroblast growth factor (FGF)-2 and platelet-derived growth factor (PDGF)-AB are a known therapeutical target in pulmonary fibrosing diseases, e.g. IPF, but there is no targeted therapy option for pulmonary fibrosis in sarcoidosis.

**Objectives:**

The aim of our study was to determine the association of these markers’ serum levels on lung function and the patients’ quality of life in a long-term follow-up of sarcoidosis patients, to provide further information for finding targeted therapy options for pulmonary sarcoidosis.

**Methods:**

54 patients with sarcoidosis underwent blood sampling, pulmonary function testing and answered the King’s Brief Interstitial Lung Disease (K-BILD) questionnaire at baseline and at three-years follow-up. Serum levels of profibrotic and angiogenic markers were assessed at baseline by enzyme-linked immunosorbent assay.

**Results:**

Between 2015 and 2018, 54 patients with biopsy proven sarcoidosis were enrolled. Throughout the observation period, there was a significant decrease in the diffusion capacity for carbon monoxide (DLCO) [%] (-6.5504 ± 13,39, p = 0.001) and forced expiratory volume in one second predicted (FEV1) [%] (-6.07 ± 12.09, p = 0.001). Patients with greater impairment of forced vital capacity (FVC) did have significantly higher serum levels of VEGF (p = 0.03) and PDGF-AB (p<0.001). The K-BILD questionnaire did not change significantly during follow-up. However, patients with worsening K-BILD scores did have significantly higher serum-levels of PDGF-AB (2.67 pg/ml ± 0.93 vs. 1.88 pg/ml ± 0.60, p = 0.004) at baseline, compared to those with unchanged or increasing K-BILD scores.

**Conclusions:**

Among patients with pulmonary sarcoidosis, baseline serum levels of VEGF and PDGF-AB were associated with pulmonary function impairment. Furthermore, PDGF-AB was associated with worsening K-BILD scores. No such association was observed for FGF-2 and TGF-ß1. VEGF and PDGF-AB may be possible prognostic and therapeutic targets in sarcoidosis as a fibrosing ILD beyond IPF.

## 1. Introduction

Sarcoidosis is a systemic inflammatory granulomatous disease most frequently affecting the lung and the mediastinal lymph nodes and can potentially involve any other organ. The disease is characterised by the formation of epithelioid-like lesions, also described as non-caseating granuloma, in the organ tissue. These granulomas may impair organ function and cause a variety of symptoms. Pulmonary involvement is observed in >90% of the patients [1].

The aetiology of sarcoidosis is still unknown. Taken together, there is a hypothesis that environmental triggers are involved, probably inhalative antigens, which trigger an excessive reaction of the immune system in people with a hereditary disposition. Pro-inflammatory cytokines maintain a state of chronic inflammation [2, 3]. This inflammatory milieu may promote fibrosis of the lung tissue.

Despite the lack of extensive knowledge regarding the genesis of pulmonary fibrosis, its impact on patients’ health and well-being is unquestioned. Lung fibrosis is one of the main causes of death in sarcoidosis [2].

The current medical treatment options are limited to immunosuppressant medication, mainly glucocorticoid therapy. Nevertheless, such therapy is rather unspecific. So far, there are no targeted treatment options available, neither for sarcoidosis nor for the accompanying lung fibrosis.

Little is known about the cause of pulmonary fibrosis, neither in sarcoidosis nor in other fibrosing lung diseases, e.g. idiopathic pulmonary fibrosis (IPF), but it may be postulated that similar pathways are involved [4].

For IPF, there are two antifibrotic therapy options available. One is nintedanib, a tyrosine kinase inhibitor (TKI), that acts as an inhibitor of the PDGF-, FGF- and VEGF receptors [5]. The other one is pirfenidone, which attenuates key TGF-β-induced signalling pathways [6]. Pirfenidone is also used on a variety of other fibrotic diseases in different organs [7].

The INBUILD trial recently showed an anti-fibrotic effect for nintedanib on lung function in different fibrosing interstitial lung diseases other than IPF, including sarcoidosis [8]. Accordingly, there is a growing awareness of antifibrotic medication possibly being effective for a variety of fibrosing pulmonary diseases besides IPF.

The aim of this study was to assess the serum levels of those four proangiogenic and profibrotic markers VEGF, TGF-β1, FGF-2 and PDGF-AB in sarcoidosis and if there is an association of these serum markers with pulmonary impairment and quality of life. Therefore, there are two primary questions addressing these two subjects.

First, is there an association between the serum markers and lung impairment, defined as reduced FVC and/or DLCO, and secondly, is there an association between the serum markers and quality of life, assessed by K-BILD questionnaire.

## 2. Methods

This study is a prospective, single-centre, longitudinal study, investigating the association of the serum levels of markers of fibrogenesis and angiogenesis on pulmonary impairment and quality of life in sarcoidosis patients.

### 2.1 Patients

54 patients with pulmonary sarcoidosis at all stages with and without extrapulmonary involvement above the age of 18 were enrolled at the pneumological outpatient department, University Hospital of Bonn (Germany), between May and December 2015. Only patients with a diagnosis that had been confirmed by biopsy were enrolled. Exclusion criteria comprised other significant pulmonary diseases, e.g. chronic obstructive pulmonary disease (COPD), lung cancer or pulmonary fibrosis of any other cause. Exclusion criteria were assessed by patients’ medical reports.

Tests taken at the time of enrolment and at three-years follow-up were in the context of a routine check-up. At enrolment, we obtained pulmonary function tests, blood tests including ACE-, sIL-2-, VEGF-, TGF-ß1-, FGF-2- and PDGF-AB- serum levels, and asked the subjects to complete the K-BILD questionnaire. The radiological stage of sarcoidosis was determined as Scadding stage by the latest chest radiograph in the patient’s medical record [1, 9]. At follow-up, we obtained pulmonary function test and the K-BILD questionnaire.

The study was approved by the local ethics committee (Ethik-Kommittee der Medizinischen Fakultät, Rheinische Friedrich-Wilhelms-Universität Bonn) and was in accordance with the Declaration of Helsinki. All participants gave informed written consent. They underwent anamnesis, peripheral blood withdrawal, pulmonary function testing, blood gas analysis and answered the K-BILD questionnaire at baseline and follow-up.

### 2.2 Pulmonary function test

Static and dynamic lung volumes [forced vital capacity (FVC), forced expiratory volume in one second (FEV1), Tiffeneau Index (FEV1/VC)] and diffusion capacity for carbon monoxide (DLCO) by single-breath method were assessed. Predicted values were calculated based on age, sex and height by the software of the pulmonary function test (Bodyplethismograph Jaeger©, Alveo-Diffusionstest Jaeger©, Wuppertal, Germany). A significant decline in FVC [L] was defined as a decline of ≥ 10%, and ≥ 15% for DLCO [%], based on the definitions from the INBUILD trial [8].

### 2.3 Blood tests

Levels of angiotensin converting enzyme (ACE) and soluble interleukin-2-receptor (sIL-2R) were assessed from patient blood samples at baseline. VEGF, TGF-ß1, FGF-2 and PDGF-AB serum levels were analysed at baseline by enzyme-linked immunosorbent assay [10] after centrifugation and preservation of the baseline blood samples in an– 80°C cooler.

### 2.4 K-BILD

Health-related quality of life was assessed by the “King’s Brief Interstitial Lung Disease (K-BILD) Health Status Questionnaire”, henceforth referred to as K-BILD, at baseline and follow-up. The K-BILD is a commonly used, validated tool to evaluate ILD health-related quality of life in ILD patients, the German translation has been validated and was used in this study [11]. 15 items evaluate four categories, namely breathlessness and activities, psychological aspects and chest symptoms. The total score after logit transformation ranges from 0–100, with 100 points indicating the best quality of life [11]. The minimal clinically significant change was defined as a change of 8 points [12].

### 2.5 Statistics

Statistical analysis was performed by using IBM SPSS Statistics 25. The significance level (α) was set to 5%, and p<0.05 was considered statistically significant. For differences between two groups at baseline, Levene’s test for equality of variances was performed, before using student’s t-test for independent variables. For the comparison of more than two groups, ANOVA was performed, whereas for post-hoc analyses, the Tukey-test was used. Comparison with follow-up data was made by using the student’s t-test for dependent variables.

Of note, some of the patient numbers (Tables 1 and 2) do not add up to 54, as a few patients did not receive complete pulmonary function testing either at baseline or follow up during routine check-up. One patient did not receive DLCO testing at baseline, so the number of patients with DLCO values in Table 1 equals 53. Furthermore, two patients did not receive FVC [L] testing at baseline, while for two other patients it is missing at follow-up. Therefore, decline of FVC [L] could only be calculated for 50 patients. This also applies to the K-BILD questionnaire (Table 4).

**Table 1:** Patients’ characteristics at baseline (n = 54).

Age	52.6 ± 11.8
Gender (male)	29 (53.7%)
Radiological stage:	
I	13 (24.1%)
II	25 (46.3%)
III	13 (24.1%)
IV	3 (5.6%)
Radiological pulmonary involvement (stages III + IV)	16 (29.6%)
Manifestation of disease	
predominantly pulmonary	31 (57.4%)
predominantly extrapulmonary	3 (5.6%)
Mixed	20 (37.0%)
DLCO	
≥ 80%	19 (15.7%)
60–80%	27 (22.3%)
40–60%	5 (4.1%)
< 40%	2 (1.7%)
FVC	
≥ 80%	39 (32.2%)
60–80%	10 (8.3%)
40–60%	3 (2.5%)
< 40%	0 (0.0%)
ACE or sIL-2R elevated	24 (44.4%)
OCS	33 (61.1%)
PDGF-AB [pg/ml]	1.99 ± 0.72
VEGF [pg/ml]	86.08 ± 41.86
FGF-2 [pg/ml]	1.82 ± 0.26
TGF-β1 [ng/ml]	23.86 ± 11.03

Data are presented as n (%) or mean ± SD.

ACE = Angiotensin converting enzyme, elevated >85 U/l. sIL2-R = soluble interleukin-2-receptor, elevated >730 U/ml. DLCO = diffusion capacity for carbon monoxide. FVC = forced vital capacity. OCS = Oral corticosteroid treatment. PDGF-AB = platelet-derived growth factor-AB. VEGF = vascular endothelial growth factor. FGF-2 = fibroblast growth factor-2. TGF- β1 = transforming growth factor-β1.

**Table 2:** Changes of lung function parameters from baseline to follow-up after three years.

	Baseline	3 years	p-value
DLCO [%]	73.6 ± 16.4	67.0 ± 18.1	0.001*
FVC [L]	3.8 ± 1.1	3.7 ± 1.1	0.451
FVC [%]	90.8 ± 20.3	90.2 ± 20.0	0.676
FEV1 [L]	3.0 ± 0.9	2.9 ± 0.9	0.055
FEV1 [%]	91.6 ± 21.5	85.6 ± 20.8	0.001*
FEV1/FVC [%]	79.7 ± 8.6	81.3 ± 10.3	0.261
Capillary pO2 [mmHg]	78.0 ± 10.6	77.6 ± 8.8	0.802
	Yes	no	
Decline of FVC [L] ≥ 10%	10 (20%)	40 (80%)	
Decline of DLCO [%] ≥ 15%	15 (28.3%)	38 (71.7%)	

Data are presented as mean ± SD. DLCO = diffusing capacity for carbon monoxide. FVC = forced vital capacity. FEV1 = forced expiratory volume in one second. FEV1/FVC = Tiffeneau-Index. pO2 = oxygen partial pressure. * = p < 0.05.

## 3. Results

### 3.1 Participants

Study participants were followed-up for three years. The patient’s characteristics at baseline are presented in Table 1.

The mean age was 52.6 ± 11.8 years, and 53.7% were male. At baseline, 51 patients (94.4%) had mixed or predominantly pulmonary involvement, three (5.6%) had predominantly extrapulmonary involvement. 24 patients (44.4%) had elevated ACE or sIL-2R serum-levels (ACE >85 U/l or sIL2-R >730 U/ml, based on local laboratory’s cut-off values), 33 (61.1%) were under oral corticosteroid treatment. Participants had a normal baseline FVC of 3.8 L ± 1.1 (90.8% ± 20.3 predicted), and a mild impairment of DLCO (73.6% ± 16.4 predicted).

### 3.2 Lung function and serum markers

**Fig 1:**
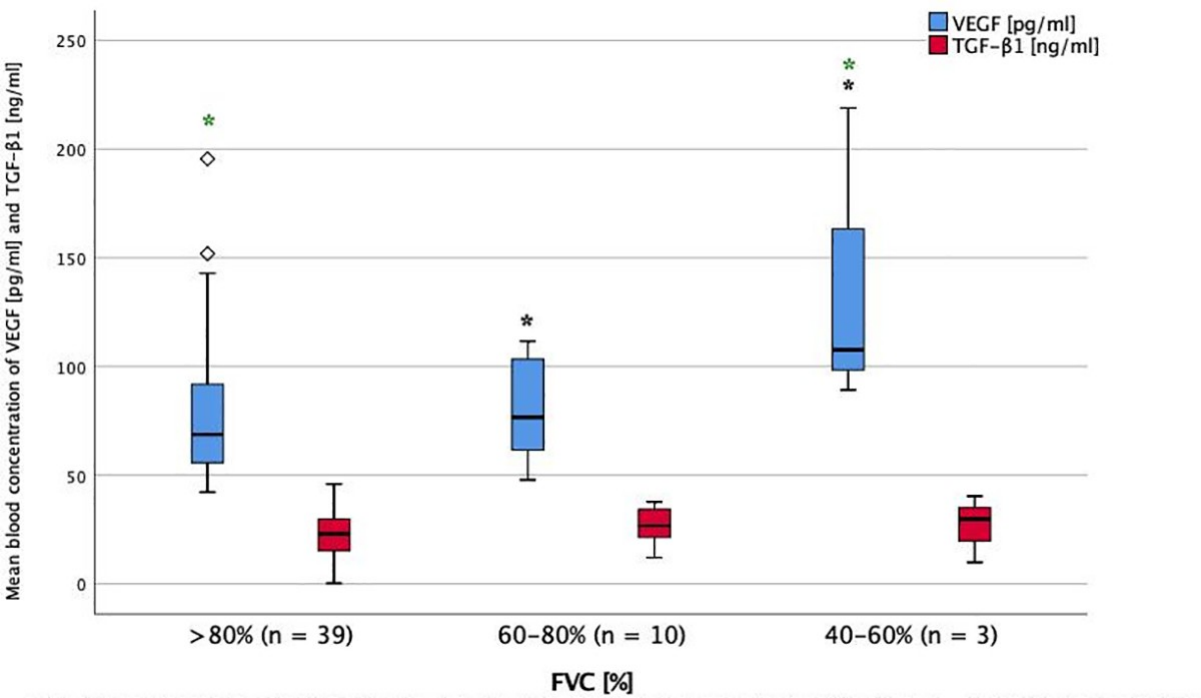
Serum levels of TGF-ß1 and VEGF in impairment of FVC at baseline. Mean blood concentration of VEGF and TGF-β1 in different stages of FVC impairment. FVC = forced vital capacity. TGF-β1 = transforming growth factor beta-1. VEGF = vascular endothelial growth factor. * = P< 0.05 (ANOVA + Tukey-test as post-hoc test for multiple comparisons). Significant difference between groups: VEGF in FVC >80% and 40–60%, VEGF in FVC 60–80% and 40–60%.

**Fig 2:**
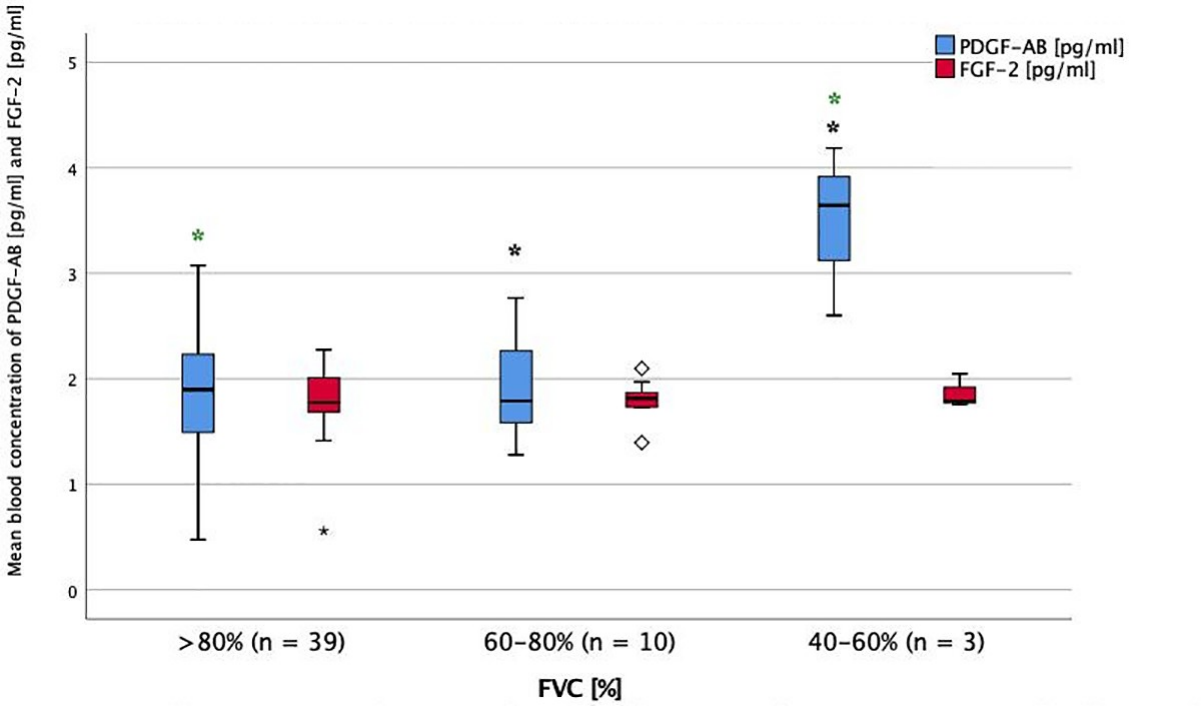
Serum levels of FGF-2 and PDGF-AB in impairment of FVC at baseline. Mean blood concentration of PDGF-AB and FGF-2 in different stages of FVC impairment. FGF-2 = fibroblast growth factor-2. FVC = forced vital capacity. PDGF-AB = platelet derived growth factor-AB. * = p < 0.05 (ANOVA + Tukey-test as post-hoc test for multiple comparisons). Significant difference between groups: PDGF-AB in FVC >80% and 40–60%, PDGF-AB in FVC 60–80% and 40–60%.

**Fig 3:**
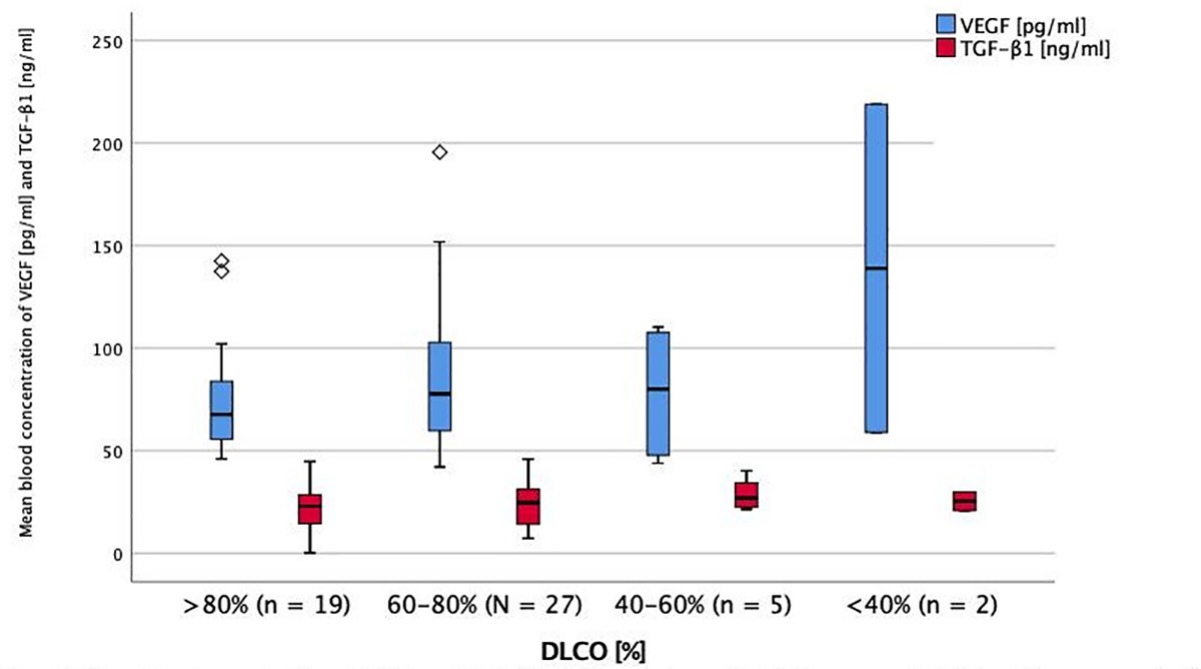
Serum levels of and TGF-ß1 and VEGF in impairment OF DLCO at baseline. Mean blood concentration of VEGF and TGF-B1 in different stages of DLCO impairment. DLCO = diffusion capacity for carbon monoxide. TGF-B1 = transforming growth factor beta-1. VEGF = vascular endothelial growth factor. * = p < 0.05 (ANOVA + Tukey-test as post-hoc test for multiple comparisons). No significant difference between groups.

**Fig 4:**
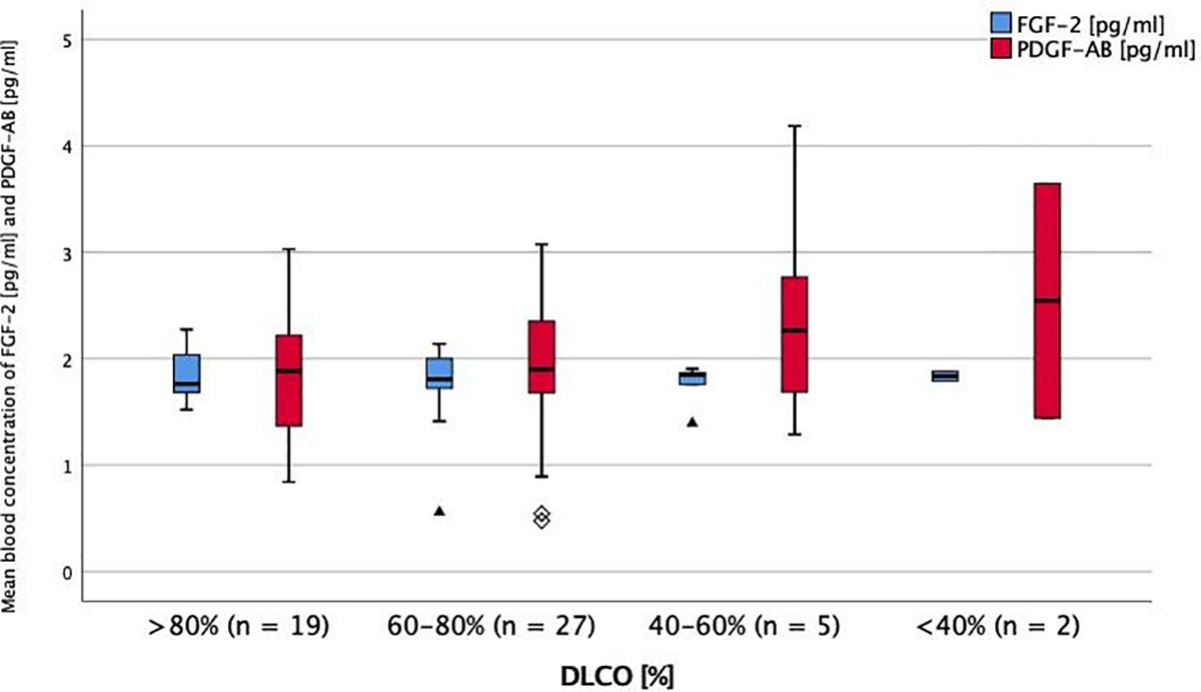
Serum levels of FGF-2 and PDGF-AB in impairment of DLCO at baseline. Mean blood concentration of FGF-2 and PDGF-AB in different stages of DLCO impairment. DLCO = diffusion capacity of carbon monoxide. FGF-2 = fibroblast growth factor-2. PDGF-AB = platelet derived growth factor-AB. * = p < 0.05 (ANOVA + Tukey-test as post-hoc test for multiple comparisons). No significant differences between the groups.

**Fig 5:**
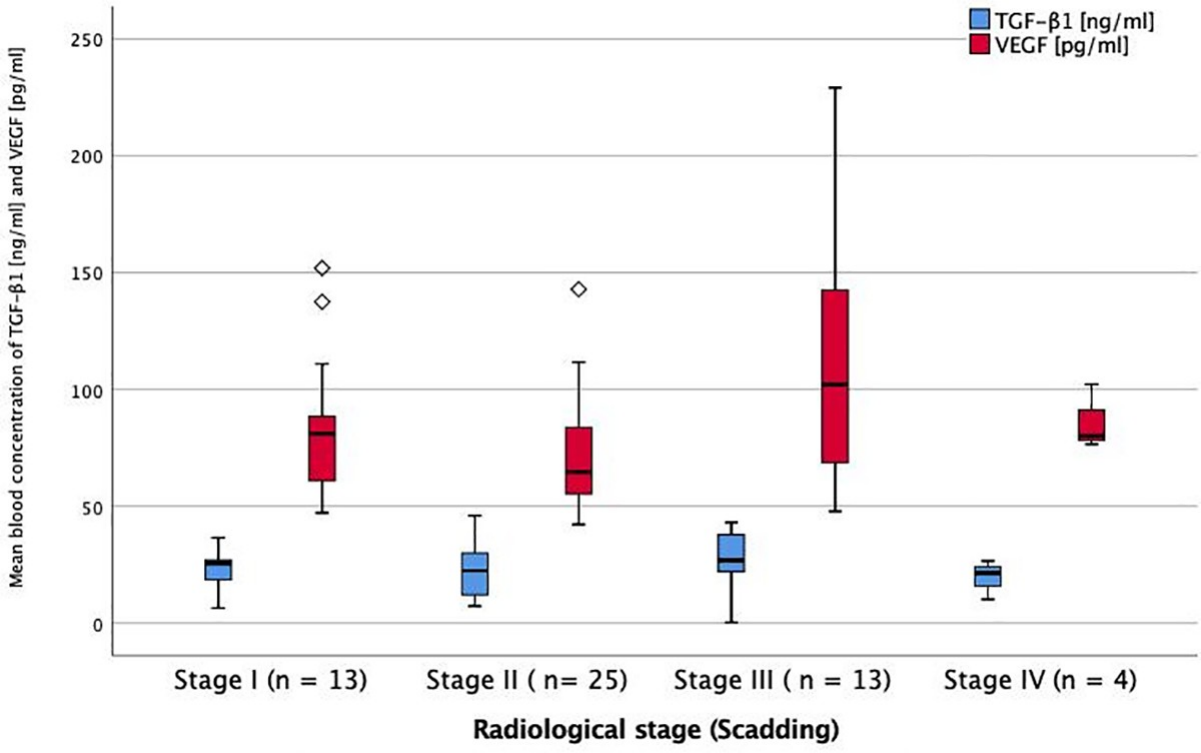
Serum levels of TGF-ß1 and VEGF in different radiological stages of sarcoidosis at baseline. Mean blood concentration of TGF-β1 and VEGF in different radiological stages of sarcoidosis. TGF-β1 = transforming growth factor beta-1. VEGF = vascular endothelial growth factor. * = p < 0.05 (ANOVA + Tukey-test as post-hoc test for multiple comparisons). No significant difference between groups.

**Fig 6:**
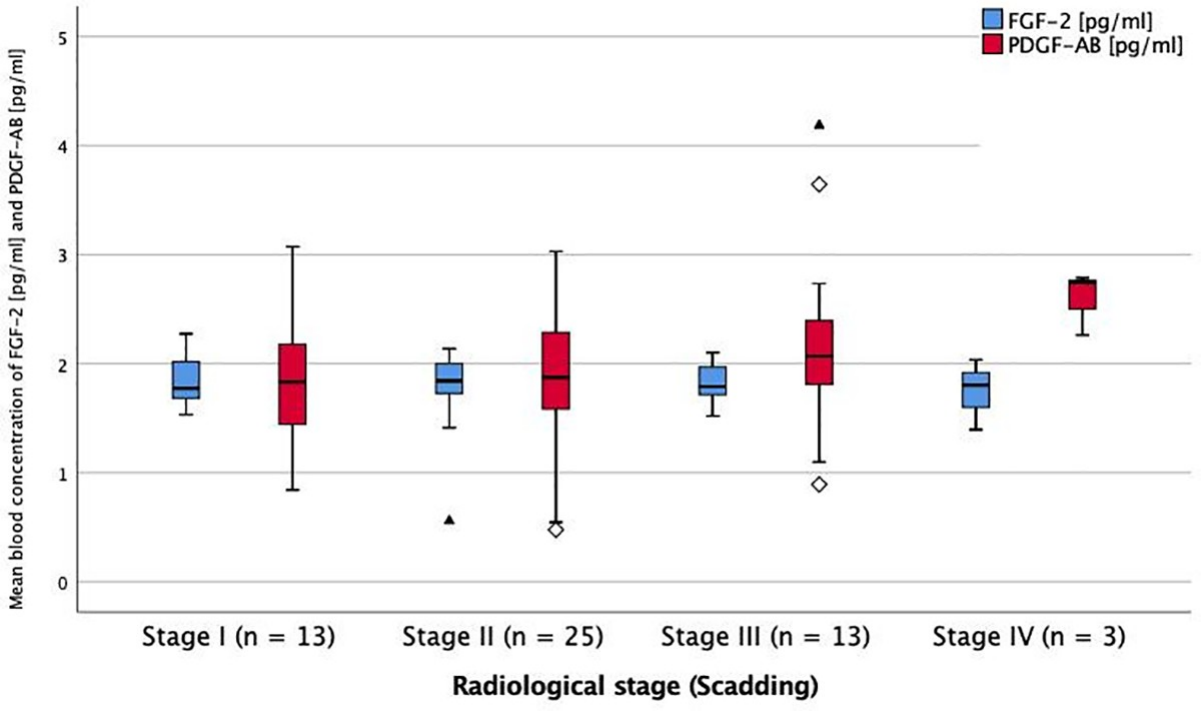
Serum levels of FGF-2 and PDGF-AB in different radiological stages of sarcoidosis at baseline. Mean blood concentration of FGF-2 and PDGF-AB in different radiological stages of sarcoidosis. FGF-2 = fibroblast growth factor-2. PDGF-AB = platelet derived growth factor-AB. * = p < 0.05 (ANOVA + Tukey-test as post-hoc test for multiple comparisons). No significant difference between groups.

The serum levels of markers of fibrosis and angiogenesis at baseline were analysed with regard to the different stages of sarcoidosis and lung function impairment. For a better understanding, these results are visually displayed in Figs 1–6.

There was an association of PDGF-AB and VEGF serum levels with lung function, as they were significantly higher in the group with the lowest FVC-values (p<0.001 and p = 0.030, Figs 1 and 2).

Even though PDGF and VEGF levels were highest in patients with major diffusion impairment, there was no significant difference between the markers’ serum levels in different stages of DLCO impairment (Figs 3 and 4).

### 3.3 Radiological stage and serum markers

Comparing the different radiological stages of sarcoidosis I-IV at baseline, classified by chest radiograph, VEGF-levels were highest at stage III and significantly higher compared to stage II (p<0.005). There were no significant differences of PDGF-AB, FGF-2 and TGF-β1 levels between the radiological stages (Figs 5 and 6).

### 3.4 ACE, sIL-2-receptor and oral corticosteroid treatment

There was no difference in the serum levels of the markers in patients with elevated angiotensin converting enzyme (ACE >85 U/l) and/or soluble interleukin-2-receptor (sIL-2-R >730 U/ml), or in patients with or without oral corticosteroid treatment (OCS).

### 3.5 Longitudinal changes

At follow-up, 10 patients (20%) had a significant decline of FVC [L], and 15 patients (28.3%) of DLCO [%]. There was a significant absolute decline in DLCO (from 73.58% ± 16.41 to 67.03% ± 18.07, p = 0.001) and in FEV1 predicted (FEV1 from 91.6% ± 21.5 to 85.6% ± 20.8, p = 0.001)). FVC (p = 0.451), FEV1/VC (p = 0.261) and capillary oxygen partial pressure (p = 0.802) did not change significantly (Table 2).

### 3.6 Health related quality of life (HRQOL)

From a maximum of 100 points, the recruited patients had a total K-BILD score of 65.7 ± 14.3 at baseline. Considering all patients, there was no significant change in K-BILD score over the course of three years (66.5 ± 15.0, p = 0.510). There was no significant change in different health domains (psychological items, breathlessness and activities, chest symptoms) (Table 3).

**Table 3:** Development of K-BILD scores from baseline to follow-up after three years.

	Baseline	3 years	p-value
K-BILD total score	65.7 ± 14.3	66.5 ± 15.0	0.510
K-BILD subscores			
Psychological aspects	65.5 ± 18.1	67.0 ± 19.7	0.362
Breathlessness and activities	59.4 ±19.9	59.2 ± 19.2	0.876
Chest symptoms	76.8 ± 19.9	76.2 ± 20.0	0.740

Score in K-BILD. Data are presented as mean ± SD.

An association of the serum markers and HRQOL could be seen, as those patients with a declining HRQOL (9.3%) at three-years follow-up did have significantly higher baseline serum-levels of PDGF-AB, compared to patients with stable or improving HRQOL results [1.89 pg/ml ± 0.59 (p = 0.004)] (Table 4).

**Table 4:** Profibrotic and angiogenic serum markers and lung function parameters in patients with declining K-BILD scores.

	K-BILD total score from baseline to follow-up		p-value
	Stable or improving (n = 39)	Declining (n = 4)	
Change in K-BILD total score	1.9 ± 6.8	- 10.1 ± 3.9	0.001*
Serum levels at baseline			
FGF-2 [pg/ml]	1.82 ± 0.28	1.65 ± 0.10	0.229
VEGF [pg/ml]	83.69 ± 38.90	122.60 ± 29.69	0.060
PDGF-AB [pg/ml]	1.89 ± 0.59	2.87 ± 1.01	0.005
TGF-β1 [ng/ml]	24.27 ± 11.40	23.28 ± 16.72	0.874
Change of lung function parameters from baseline to follow-up			
DLCO [%]	- 8.0 ± 11.9	-14.3 ± 7.1	0.514
FVC [L]	0.0 ± 0.5	- 0.3 ± 0.6	0.314
FVC [%]	0.4 ± 11.9	- 4.3 ± 13.8	0.461
FEV1 [L]	- 0.1 ± 0.4	- 0.2 ± 0.2	0.479
FEV1 [%]	- 5.8 ± 14.0	- 8.2 ± 3.5	0.733
FEV1/FVC [%]	1.4 ± 9.4	- 1.8 ± 11.3	0.522

Data are presented as mean ± SD.

PDGF-AB = platelet-derived growth factor-AB. VEGF = vascular endothelial growth factor. FGF-2 = fibroblast growth factor-2. TGF- β1 = transforming growth factor-β1. DLCO = diffusing capacity for carbon monoxide. FVC = forced vital capacity. FEV1 = forced expiratory volume in one second. FEV1/FVC = Tiffeneau-Index.

Declining K-BILD Score = decline of ≥ 8 points.

* = p < 0.05.

The change of lung function parameters from baseline to follow-up did not differ significantly between patients with a stable/improving K-BILD total score and those with declining scores (Table 4).

## 4. Discussion

The present study in sarcoidosis patients under real-life conditions showed an association of lung function impairment with higher serum levels of profibrotic and proangiogenic PDGF-AB and VEGF. Furthermore, worsening HRQOL was associated with higher serum levels of PDGF-AB. For FGF-2 and TGF-β1, no such associations could be shown.

VEGF and PDGF-AB serum levels at baseline were highest in patients with the lowest FVC-values.

The current evidence is not conclusive regarding VEGF levels in sarcoidosis.

One the one hand, in one study investigating several serum markers, VEGF concentrations did not differ significantly between patients with sarcoidosis, IPF or controls. No correlation between VEGF serum concentrations and pulmonary function parameters was observed [13]. However, there were no stage IV sarcoidosis patients enrolled, which would show the most significant impairment of lung function.

On the other hand, another study showed higher serum levels of VEGF in patients with severe sarcoidosis who received corticosteroid treatment. The authors postulated serum VEGF as a marker for the onset and prognosis of sarcoidosis [14].

A further study could show higher VEGF gene expression in cells from broncho-alveolar lavage (BAL) from sarcoidosis patients with abnormal spirometry [15]. The results of these two studies are more in line with our results. The main difference between the latter study and our one is that the markers were measured from local samples (BAL), while we measured circulatory markers. A major advantage of measuring markers from peripheral blood is their accessibility. They can possibly be used for diagnostic and/or prognostic matters with much lower risk than taking BAL or tissue samples. There is one study approaching this issue, where the concentrations of VEGF were compared between BAL fluid and serum for 65 sarcoidosis patients, and they were elevated accordantly [16]. However, it is unknown to which extent inferences can be drawn from circulatory markers to local markers and vice versa, so this point remains of interest for further research.

While an association between two serum markers and pulmonary function parameters was seen, the connections between the serum markers and pulmonary involvement defined by chest x-ray are less conclusive. The fact that VEGF levels were significantly higher in radiological stage III than stage II may indicate an association of VEGF levels and pulmonary involvement. But as there is no significant difference of VEGF levels between stage I or II and stage IV, and no linear relation from stages I to IV, it may well be due to chance.

Compared to data on VEGF in sarcoidosis, data on PDGF and FGF is scarce.

For example, Du et al. showed no difference comparing serum levels of PDGF-BB in sarcoidosis patients, tuberculosis patients and healthy controls [17].

One recent study analysed VEGF, PDGF and FGF concentrations in lung tissue samples of patients with fibrotic lung diseases that underwent lung transplantation, including six sarcoidosis patients [18]. Concentrations were significantly increased, compared to donor lungs. Here again, markers were obtained from tissue samples, not from serum.

As nintedanib acts as an inhibitor of the PDGF-, FGF- and VEGF receptors, the authors hypothesised that nintedanib treatment should also be effective in other fibrosing lung diseases than IPF. Interestingly, no correlations with lung function parameters could be made [18].

The INBUILD trial finally showed the anti-fibrotic effect of nintedanib in a variety of fibrosing interstitial lung diseases other than IPF, including sarcoidosis [8].

While we could not show a significant association of TGF-β1 serum levels and pulmonary sarcoidosis, other studies on TGF-β1 show conflicting results. For example, one study including 57 sarcoidosis patients measured elevated serum levels of TGF-β1 and VEGF, compared to healthy controls. This difference could not be found in BAL. There was neither a difference of serum levels between patients with and without lung parenchymal involvement, nor between patients with normal and abnormal lung function [19]. On the other hand, Salez and colleagues observed elevated TGF-β1 levels in BAL of sarcoidosis patients with altered lung function results [20].

Different genetic variations of TGF- β subclasses have been suggested to play a role in the fibrotic course of pulmonary sarcoidosis [21], which is a possible explanation for contrary findings and highlights the need for further research on TGF- β and the differentiation of TGF-β subclasses.

Besides the fact that there is only limited conclusive data available for the serum markers, there is another important difficulty and limitation concerning studies investigating pulmonary fibrosis in sarcoidosis. Only a small percentage of sarcoidosis patients present a progressively fibrosing phenotype [22]. We did observe a significant decline of DLCO and FEV1 predicted in this patient collective, but there was no significant decline of FVC during follow-up, although FVC is an essential element for the definition of a progressive course of fibrotic lung disease and was the primary endpoint in the INBUILD and INPULSIS trials [8, 23]. Furthermore, there were only three patients included at stage IV and a total of 16 patients at stages III and IV at baseline. While patients presenting milder radiological stages at baseline may also develop progressively fibrosing disease with a decline in lung function, observations made in advanced radiological stages may be more robust. Additionally, the course of three years may be too short to see a significant progression, especially from patients with mild pulmonary involvement, or from patients already under immunosuppressant treatment. This is reflected in the small number of patients in our study that developed a relevant decline in FVC ≥ 10% over the three-years follow-up period (n = 10).

Therefore, bigger and multicentric studies are needed to acquire a more representative patient collective. In line with this, the results of an on-going study investigating the impact of pirfenidone on progressive fibrotic sarcoidosis by Baughman et al. will be of interest [24].

Further methodical limitations of our study are worth mentioning. For example, the inclusion of only biopsy proven disease decreases external validity. Still, most patients with pulmonary involvement undergo biopsy, and patients diagnosed clinically upon syndrome, e.g. Loefgrens, have a low risk to develop pulmonary involvement.

Further on, we are aware of the lack of a control group, especially for the markers’ serum levels. A comparison with healthy control or sarcoidosis patients without pulmonary involvement could give further insights on the association and relation of the serum markers with pulmonary involvement and/or disease progression. If the serum markers were significantly different from a control population, they could be considered as biomarkers indicating a progressive disease and underline the need for treatment. Furthermore, it could be analysed whether patients with higher serum markers respond better to a specific therapy and it could possibly be used as a biomarker for the effectiveness of such therapy. This study gives an idea which markers may be worth further research. At this point, data is too scarce to use any of these serum markers as biomarkers in a clinical setting.

We used the K-BILD as a validated tool to evaluate health-related quality of life in ILD patients. The change in K-BILD scores from baseline was below the minimal clinically significant change of 8 points [12]. Accordingly, the health-related quality of life did not change significantly.

Interestingly, patients with clinically significant worsening K-BILD results did show significantly higher serum levels of PDGF-AB at baseline than patients with stable or improving K-BILD scores. This was not reflected in a change of lung-function parameters, which did not differ between these two groups. This indicates that the decline in quality of life in this patient collective is not, or not only, caused by worsening lung function or disease progression. Atkins et al. investigated fatigue in sarcoidosis and reported an association between fatigue and poorer quality of life scores measured by, among others, the K-BILD questionnaire. However, fatigue was not associated with a decline in pulmonary function parameters [25]. As fatigue is a common symptom among sarcoidosis patients, it is hard to distinguish between a reduction in quality of life due to fatigue or pulmonary disease progression.

However, given the small number of patients with significantly declining K-BILD scores, the significance may be due to chance.

In summary, among patients with pulmonary sarcoidosis, baseline serum levels of VEGF and PDGF-AB were associated with lung function impairment. PDGF-AB was associated with a loss of health-related quality of life. These mediators may be possible prognostic and/or therapeutic targets in sarcoidosis as a fibrosing pulmonary disease beyond IPF.

## Supporting information

S1 Dataset (XLSX)

S1 File (PDF)
