# Supplementary material for: Association of proangiogenic and profibrotic serum markers with lung function and quality of life in sarcoidosis
Source: PLoS One. 2021 Feb 22;16(2):e0247197. doi: 10.1371/journal.pone.0247197 (PMC7899331; doi:10.1371/journal.pone.0247197)
Supplement: S1 File — (PDF) [file pone.0247197.s002.pdf]

King's Kurzfragebogen zur ILD (K-BILD)

Dieser Fragebogen dient der Einschätzung, wie stark Ihre Lungenerkrankung verschiedene Aspekte Ihres Lebens beeinträchtigt. Lesen Sie jede Frage sorgfältig durch und antworten Sie, indem Sie diejenige Antwort UMKREISEN, die am besten auf Sie zutrifft. Beantworten Sie bitte ALLE Fragen so ehrlich wie möglich.

ANGABEN ZUM PATIENTEN/ZUR PATIENTIN:

Name: ....

Datum: ....

Copyright by Prof. Dr. M. Kreuter, Thoraxklinik, University of Heidelberg and Dr Birring Surinder , KING'S COLLEGE HOSPITAL NHS FOUNDATION TRUST
Funded by Lungenfibrose e.V.

1. In den letzten 2 Wochen hatte ich Atemnot beim Treppensteigen oder Bergaufgehen:

1. Jedes Mal
2. Fast immer
3. Oft
4. Manchmal
5. Gelegentlich
6. Fast nie
7. Nie

2. In den letzten 2 Wochen hatte ich aufgrund meiner Lungenerkrankung ein Engegefühl in meiner Brust:

1. Die ganze Zeit
2. Den größten Teil der Zeit
3. Einen großen Teil der Zeit
4. Zeitweise
5. Selten
6. Fast nie
7. Nie

3. Haben Sie sich in den letzten 2 Wochen Sorgen darüber gemacht, wie ernst Ihre Lungenbeschwerden sind?

1. Die ganze Zeit
2. Den größten Teil der Zeit
3. Einen großen Teil der Zeit
4. Zeitweise
5. Selten
6. Fast nie
7. Nie

4. Haben Sie es in den letzten 2 Wochen vermieden, Dinge zu tun, bei denen Sie Atemnot bekommen?

1. Die ganze Zeit
2. Den größten Teil der Zeit
3. Einen großen Teil der Zeit
4. Zeitweise
5. Selten
6. Fast nie
7. Nie

5. Haben Sie in den letzten 2 Wochen das Gefühl gehabt, Ihre Lungenprobleme unter Kontrolle zu haben?

1. Nie
2. Fast nie
3. Selten
4. Zeitweise
5. Einen großen Teil der Zeit
6. Den größten Teil der Zeit
7. Die ganze Zeit

6. Haben Sie sich aufgrund Ihrer Lungenbeschwerden in den letzten 2 Wochen niedergeschlagen gefühlt oder waren Sie davon genervt?

1. Die ganze Zeit
2. Den größten Teil der Zeit
3. Einen großen Teil der Zeit
4. Zeitweise
5. Selten
6. Fast nie
7. Nie

7. In den letzten 2 Wochen hatte ich den Drang tief einatmen zu müssen, quasi „Lufthunger“:

1. Die ganze Zeit
2. Den größten Teil der Zeit
3. Einen großen Teil der Zeit
4. Zeitweise
5. Selten
6. Fast nie
7. Nie

8. Aufgrund meiner Lungenerkrankung habe ich mich in den letzten 2 Wochen besorgt gefühlt:

1. Die ganze Zeit
2. Den größten Teil der Zeit
3. Einen großen Teil der Zeit
4. Zeitweise
5. Selten
6. Fast nie
7. Nie

Copyright by Prof. Dr. M. Kreuter, Thoraxklinik, University of Heidelberg and Dr Birring Surinder , KING'S COLLEGE HOSPITAL NHS FOUNDATION TRUST
Funded by Lungenfibrose e.V.

9. Wie oft haben Sie in den letzten 2 Wochen ein pfeifendes Atemgeräusch (Keuchen) wahrgenommen?

1. Die ganze Zeit
2. Den größten Teil der Zeit
3. Einen großen Teil der Zeit
4. Zeitweise
5. Selten
6. Fast nie
7. Nie

10. Wie viel Zeit in den letzten 2 Wochen hatten Sie das Gefühl, dass Ihre Lungenerkrankung schlimmer wird?

1. Die ganze Zeit
2. Den größten Teil der Zeit
3. Einen großen Teil der Zeit
4. Zeitweise
5. Selten
6. Fast nie
7. Nie

11. Hat Ihre Lungenerkrankung in den letzten 2 Wochen Ihre Arbeit oder alltäglichen Verrichtungen beeinträchtigt?

1. Die ganze Zeit
2. Den größten Teil der Zeit
3. Einen großen Teil der Zeit
4. Zeitweise
5. Selten
6. Fast nie
7. Nie

12. Haben Sie in den letzten 2 Wochen damit gerechnet, dass Ihre Lungenerkrankung schlimmer wird?

1. Die ganze Zeit
2. Den größten Teil der Zeit
3. Einen großen Teil der Zeit
4. Zeitweise
5. Selten
6. Fast nie
7. Nie

13. Wie sehr hat Ihre Lungenerkrankung Sie in den letzten 2 Wochen daran gehindert, gewöhnliche Dinge wie z. B. den Lebensmitteleinkauf zu tragen?

1. Die ganze Zeit
2. Den größten Teil der Zeit
3. Einen großen Teil der Zeit
4. Zeitweise
5. Selten
6. Fast nie
7. Nie

14. Haben Sie in den letzten 2 Wochen aufgrund Ihrer Lungenerkrankung öfter an Ihr Lebensende gedacht?

1. Die ganze Zeit
2. Den größten Teil der Zeit
3. Einen großen Teil der Zeit
4. Zeitweise
5. Selten
6. Fast nie
7. Nie

15. Hat Ihre Lungenerkrankung bei Ihnen zu einer finanziellen Schlechterstellung geführt?

1. Hochgradig
2. Deutlich
3. Ziemlich
4. Mäßig
5. Gering
6. Kaum
7. Nein

Vielen Dank für das Ausfüllen dieses Fragebogens.

Copyright by Prof. Dr. M. Kreuter, Thoraxklinik, University of Heidelberg and Dr Birring Surinder , KING'S COLLEGE HOSPITAL NHS FOUNDATION TRUST
Funded by Lungenfibrose e.V.
